# Global research trends in immunotherapy for glioma: a comprehensive visualization and bibliometric analysis

**DOI:** 10.3389/fendo.2023.1273634

**Published:** 2023-10-05

**Authors:** Hong-yu Zhang, Han-yong Yu, Guo-xu Zhao, Xin-zhan Jiang, Ge Gao, Bao-jian Wei

**Affiliations:** ^1^ Department of Neurosurgery, Harbin Medical University, Harbin, China; ^2^ Department of Medical Imaging, Mudanjiang Medical University, Mudanjiang, China; ^3^ Department of Gastrointestinal Surgery, Linyi People’s Hospital, Linyi, China; ^4^ School of Nursing, Shandong First Medical University & Shandong Academy of Medical Sciences, Taian, Shandong, China

**Keywords:** glioma, immunotherapy, bibliometric, CAR-T, VOSviewer, Citespace

## Abstract

**Background:**

Glioma is a prevalent and lethal brain malignancy; despite current treatment options, the prognosis remains poor. Therefore, immunotherapy has emerged as a promising therapeutic strategy. However, research trends and hotspots in glioma immunotherapy have not been systematically analyzed. This study aimed to elucidate global research trends and knowledge structures regarding immunotherapy for glioma using bibliometric analysis.

**Methods:**

Publications related to immunotherapy for glioma from 2000-2023 were retrieved from Web of Science Core Collection database (WoSCC). We conducted quantitative analysis and visualization of research trends using various tools, including VOSviewer (1.6.18), CiteSpace (5.7 R3), Microsoft Charticulator, and the Bibliometrix package in R.

**Results:**

A total of 4910 publications were included. The number of annual publications exhibited an obvious upward trend since 2019. The USA was the dominant country in terms of publication output and centrality. *Frontiers in Immunology* published the most articles. Harvard Medical School ranked first in productivity among institutions. Sampson, John H. Ph.D. is the most prolific author in the field with 88 articles and a total of 7055 citations. *Clinical Cancer Research* has the largest total number and impact factor. Analysis of keywords showed immunotherapy, glioblastoma, immunotherapy, and clinical trials as hot topics. The tumor microenvironment, cell death pathways, chimeric antigen receptor engineering, tumor-associated macrophages, and nivolumab treatment represent indicating shifts in the direction of future glioma immunotherapy development.

**Conclusion:**

This bibliometric analysis systematically delineated global landscapes and emerging trends in glioma immunotherapy research. This study highlighted the prominence of Chimeric Antigen Receptor T-cell (CAR-T), Programmed Death-1 (PD-1), and nivolumab in current glioma immunotherapy research. The growing emphasis on specific neoantigens and prognostic tumor markers suggests potential avenues for future exploration. Furthermore, the data underscores the importance of strengthened international collaboration in advancing the field.

## Graphical Abstract

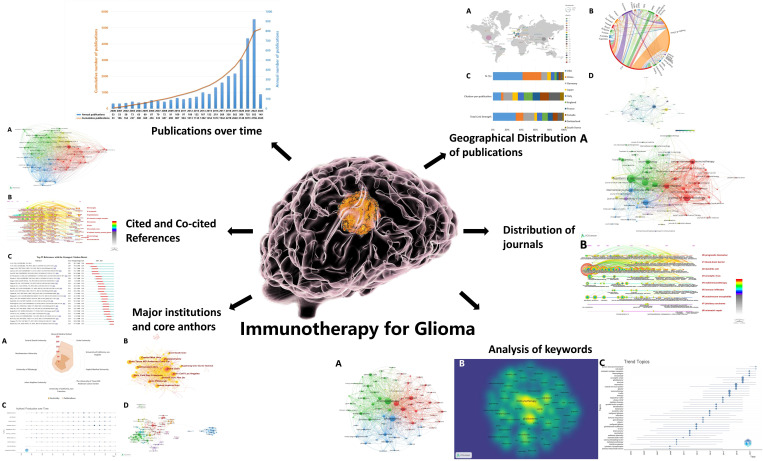

## 1 Introduction

Glioma, the most frequently occurring and fatal malignancy of the brain (1–3), continues to represent a significant clinical challenge for physicians and researchers. Gliomas, accounting for roughly 80% of all malignant brain tumors (4), impose a significant global burden with approximately 190,000 (5) new cases annually. Recent years have witnessed a surge in glioma incidence worldwide, making it a pressing public health concern. Despite the current treatment options, including surgery (6), chemotherapy (7), and radiation therapy (8), the prognosis for glioma patients remains unsatisfactory, with a 5-year survival rate of less than 10% for patients with glioblastoma (9). Glioma therapy presents several challenges: Immunosuppressive Tumor Microenvironment: Gliomas create an immunosuppressive microenvironment that hinders immune cell functionality. Tumor Heterogeneity: Tumors exhibit both inter-tumoral and intra-tumoral heterogeneity, necessitating personalized immunotherapies targeting the unique mutations within each patient’s glioma. Absence of Predictive Biomarkers: Further research is imperative to discover biomarkers that can effectively predict patient responses to specific immunotherapies. Therefore, finding effective therapies for glioma has become a major research focus in the field of oncology.

Immunotherapy, as a promising treatment modality for various cancers, has also shown potential in glioma therapy (10, 11). In recent years, the application of immunotherapy in glioma treatment has been widely explored. Immune checkpoint inhibitors (12), chimeric antigen receptor T-cell (CAR-T) therapy (13), cytokine therapy (14) and tumor vaccines (15) are the most widely studied immunotherapy strategies for glioma. Immunotherapy for glioma utilizes diverse approaches to augment the immune system’s capacity to target and eliminate tumor cells. Importantly, immune checkpoint inhibitors have demonstrated substantial efficacy in select glioma patients by inhibiting the negative regulation of T cells to augment the immune response against cancer cells (16–18). The anti-tumor properties of these inhibitors are strongly influenced by type I interferon and may also be mediated through the activation of the Stimulator of Interferon Genes (STING). These findings are paving the way for the development of innovative intervention strategies that leverage checkpoint inhibitors to enhance M1-associated anti-tumor immunity. In the treatment of glioma, immune checkpoint inhibitors have been widely studied and used. Current studies have shown that immune checkpoint inhibitors are highly effective in the treatment of some refractory gliomas and can significantly prolong the survival of patients. For example, some clinical trials have shown that the use of Programmed Death-1 (PD-1) and Programmed Death-Ligand 1 (PD-L1) inhibitors in the treatment of patients with refractory gliomas can prolong the survival of a subset of patients. However, the efficacy of immunotherapy in glioma is still limited and requires further investigation.

In the biomedical field, bibliometrics is mainly used to evaluate research performance (19), such as co-occurrence analysis and citation analysis techniques. In the healthcare field, bibliometrics is mainly used to measure the impact and importance of research articles, to help researchers predict future research trends and focus on research priorities. Glioma immunotherapy is an important new technology, but its research direction, development trends, and research hotspots have not yet been analyzed by bibliometrics. Therefore, we conducted a comprehensive bibliometric analysis, summarizing systematically the literature related to tumor immunotherapy in recent decades. Through determinations such as distribution by decade, geography, publication sources, authors, and anthologies, we gained an in-depth understanding of the research trends and public interest in Glioma immunotherapy. In addition, we identified co-occurring authors, organizations, and keywords and evaluated the research trends and public interest in glioma immunotherapy from the perspective of bibliometrics. The results of this study will provide insights into the research trends, potential hotspots, and frontiers of glioma immunotherapy. The findings of this study will also be helpful for future research, as they will provide a comprehensive understanding of the current status and future prospects of glioma immunotherapy.

## 2 Methods

### 2.1 Data sources and search strategies

The bibliographic data analyzed were sourced from the comprehensive Web of Science Core Collection (WOSCC) (20), which encompasses the Science Citation Index Expanded and Social Science Citation Index. A comprehensive systematic search strategy was carefully devised, which was restricted to English language articles and reviews in order to conduct a thorough literature search on immunotherapy in gliomas. The search strategy consisted of the following components: [TS=(ipilimumab) OR TS=(pembrolizumab) OR TS=(nivolumab) OR TS=(tremelimumab) OR TS=(immune checkpoint blockade) OR TS=(*immunotherapy) OR TS=(vaccine) OR TS=(CAR-T) OR TS=(immune checkpoint inhibitor) OR TS=(PD-1) OR TS=(PD-L1) OR TS=(CTLA-4) OR TS=(LAG-3)) AND (TS= (glioma*) OR TS=(neurolipocytoma) OR TS=(neurospongioma) OR TS=(neuroglioma*) OR TS=(glioblastoma*) OR TS=(GBM) OR TS=(gliosarcoma) OR TS=(astrocytoma)].

**Figure 1:**
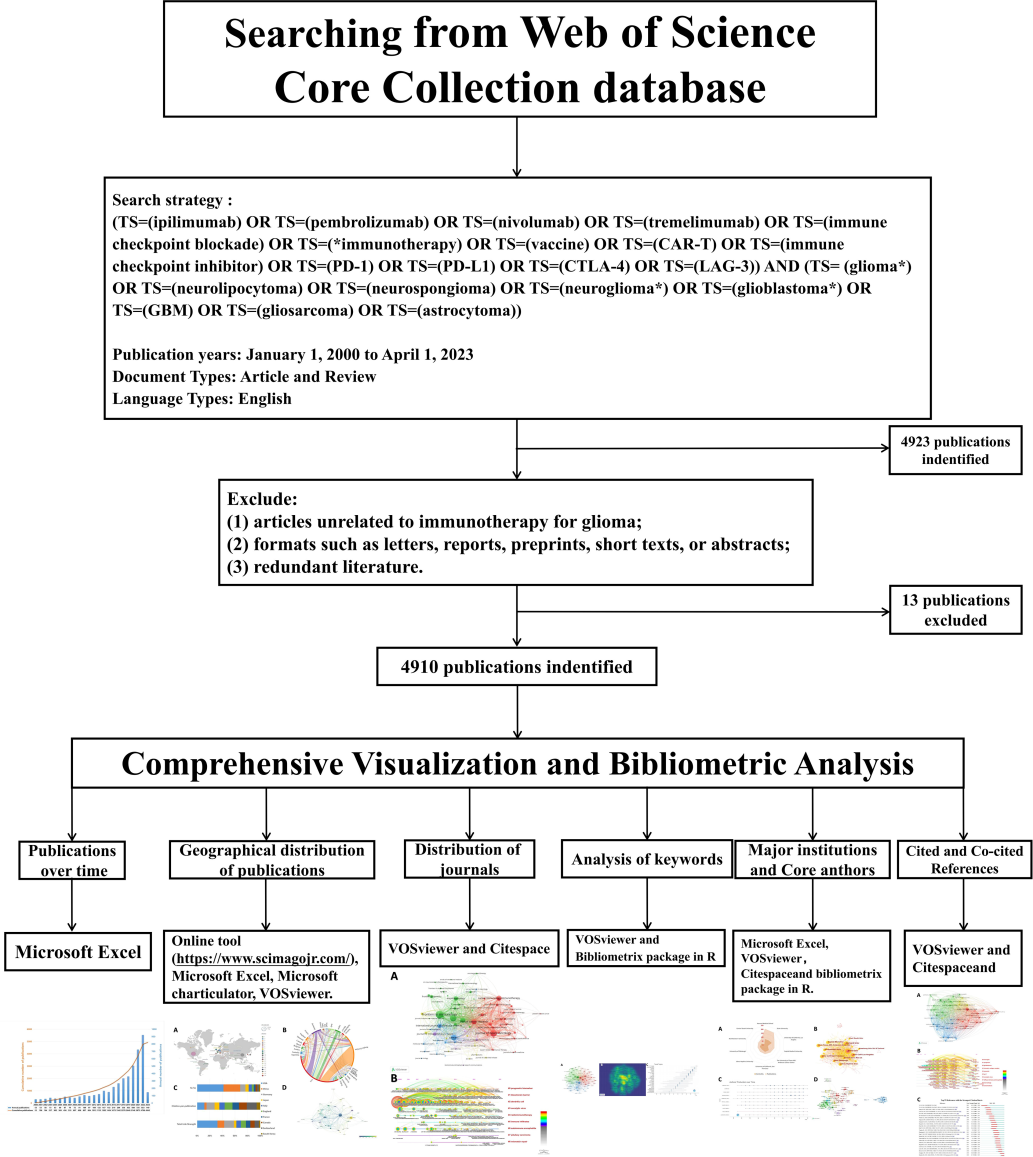
Detailed search flow chart depicts the steps in the identification and screening of papers for this bibliometric analysis. The search spanned publications from 2000 to 2023, and only documents published in English were included. Citespace software was utilized to eliminate duplicate records.

The criteria for including sources in this study were (1): a literature search period spanning from January 1, 2000 to April 1, 2023 (2); the selected literature types were “articles” and “reviews” (3); the chosen language was English. In addition, the exclusion criteria comprised (1): articles unrelated to Chinese musculoskeletal disease movement (2); formats such as letters, reports, preprints, short texts, or abstracts; and (3) redundant literature. The bibliometric process is depicted in **Figure 1**. Finally, 4910 documents were retrieved from the WOSCC.

### 2.2 Data extraction and analysis

To analyze publications related to immune checkpoint inhibitors for glioma, we conducted a bibliometric analysis, visualizing various characteristics such as distribution by year of publication, country/region, organization, journal, core authors, keywords, and key references. Our analysis employed multiple software tools: VOSviewer 1.6.18, Citespace 5.7R3, the online tool from Scimago Journal & Country Rank (https://www.scimagojr.com/), Microsoft Excel, Microsoft Charticulator, and the bibliometrix package in R (21). Specifically:

Microsoft Excel plotted the temporal distribution of both annual and cumulative publications.

Geographical publication distribution was analyzed using the Scimago online tool, Microsoft Excel, Microsoft Charticulator, and VOSviewer 1.6.18.

Analysis of major institutions and core authors involved Microsoft Excel, VOSviewer 1.6.18, the bibliometrix package in R, and Citespace 5.7R3.

Journal distribution, keyword analysis cited and co-cited reference analyses were undertaken with VOSviewer 1.6.18 and Citespace 5.7R3.

In the visual network maps, each node denotes a specific parameter such as country, institution, or keyword. The size of each node is proportional to its parameter weight, with larger nodes representing heavier weights. Both nodes and connecting lines are colored according to their assigned cluster. The distance between nodes signifies the relatedness of their co-authorship or co-citation links, while thicker lines indicate greater link strength.

### 2.3 Software and figure generation

#### 2.3.1 Figure generation by VOSviewer

We employed VOSviewer (version 1.6.18) for the creation of network visualizations.

##### 2.3.1.1 Data preparation and import

Data Source: Data was sourced from Web of Science in the form of plain text files.

Data Formatting: The data was formatted according to VOSviewer’s input requirements, often as a CSV or TXT file.

Import into VOSviewer: Through the ‘File’ -> ‘Import’ menu, the prepared data files were imported into the software.

##### 2.3.1.2 Setting parameters and thresholds

Minimum Documents per Author: For the author analysis, a minimum threshold of 12 publications per author was set. This was chosen to focus on authors with substantial contributions to the field.

Keyword Occurrence: For keyword analysis, a minimum occurrence threshold of 90 was established to include only the most relevant and frequently occurring terms.

##### 2.3.1.3 Network visualization

Visualization Type: The type of visualization (e.g., network, overlay, density) was selected based on the specific research questions being addressed.

Layout: The layout settings were adjusted for optimal visibility and interpretability. For example, we used the ‘LinLog’ layout for co-authorship networks.

Clustering: The software’s built-in clustering algorithm was applied to group closely related nodes.

##### 2.3.1.4 Customization and annotation

Node and Edge Customization: Nodes were color-coded based on clusters, and edge thickness was adjusted to represent the strength of the relationship.

Labels: Labels were added for the most significant nodes (e.g., authors with the highest number of co-authorships or keywords with the highest occurrences).

##### 2.3.1.5 Exporting figures

Format: The figures were exported as high-resolution PNG files for inclusion in the manuscript.

**Supplementary Data**: The underlying data and settings files (.vsn and.map files) were saved for future reference and are available upon request for reproducibility.

#### 2.3.2 Figure generation by Citespace

We employed CiteSpace (version 5.7.R3) for various bibliometric analyses.

##### 2.3.2.1 Data preparation and import

Data Source: Bibliographic data was extracted from databases like Web of Science and Scopus in compatible formats such as BibTeX or RIS.

Import into CiteSpace: The files were imported into CiteSpace through the ‘File’ -> ‘Import’ menu.

##### 2.3.2.2 Setting parameters and time slices

Time Slices: Data was divided into time slices of specific durations (e.g., one-year slices from 2000 to 2020) to analyze trends over time.

Thresholds: Specific thresholds for nodes and links were set based on the study’s requirements. For example, we set a minimum of n citations for nodes in the co-citation network.

##### 2.3.2.3 Network generation and analysis

Type of Network: The type of network (e.g., co-citation, co-authorship, etc.) was selected based on the research questions.

Node and Link Types: Depending on the analysis, we selected appropriate node types (e.g., authors, journals, countries) and link types (e.g., citations, co-citations).

Pruning: Unnecessary or less informative nodes and links were pruned using built-in functions like Pathfinder and Burst Detection.

##### 2.3.2.4 Visualization and customization

Layout Algorithms: Algorithms like Force Atlas or Fruchterman-Reingold were used for network layout.

Node Customization: Nodes were color-coded or sized based on specific metrics like centrality or citation count.

Labels: Significant nodes were labeled for easier interpretation.

##### 2.3.2.5 Exporting figures and data

Export Format: High-resolution figures were exported in PNG or JPEG format for inclusion in the manuscript.

Data Export: Raw data and network files were also saved in formats like GML for future reference or additional analyses.

#### 2.3.3 Figure generation by Scimago Journal & Country Rank

We utilized Scimago Journal & Country Rank (ScimagoJR) to generate figures for Geographical publication distribution.

##### 2.3.3.1 Data collection and preparation

Data Source: The primary source of our data was the Scimago Journal & Country Rank website (URL: https://www.scimagojr.com/).

Data Extraction: We manually downloaded the relevant datasets or used available APIs, focusing on specific fields and years based on our research objectives.

##### 2.3.3.2 Criteria and parameters

Fields and Categories: We restricted our analysis to specific fields or categories (e.g., Medicine, Computer Science) to align with our research questions.

Time Range: We selected a specific time range (e.g., 2010-2020) for a longitudinal perspective.

Metrics: The key metrics we analyzed included the Scimago Journal Rank (SJR), Source Normalized Impact per Paper (SNIP), and H-index.

##### 2.3.3.3 Data analysis and visualization

Data Sorting: The data was sorted based on specific metrics like SJR or H-index to identify top journals or countries.

Data Aggregation: For some analyses, we aggregated data at the country or field level.

Visualization Tools: We used Excel or specific data visualization tools to generate bar graphs, line charts, or heat maps based on the sorted and aggregated data.

##### 2.3.3.4 Figure customization

Labels and Legends: All figures included appropriate labels, titles, and legends to improve readability and interpretability.

Color Coding: Where applicable, color-coding was applied to differentiate between different fields, years, or metrics.

##### 2.3.3.5 Data and figure export

Export Format: Figures were exported in high-resolution PNG or SVG formats for inclusion in the manuscript.

Data Archiving: The extracted data and generated figures were archived and are available upon request for research reproducibility.

## 3 Results

### 3.1 Publications over time

**Figure 2:**
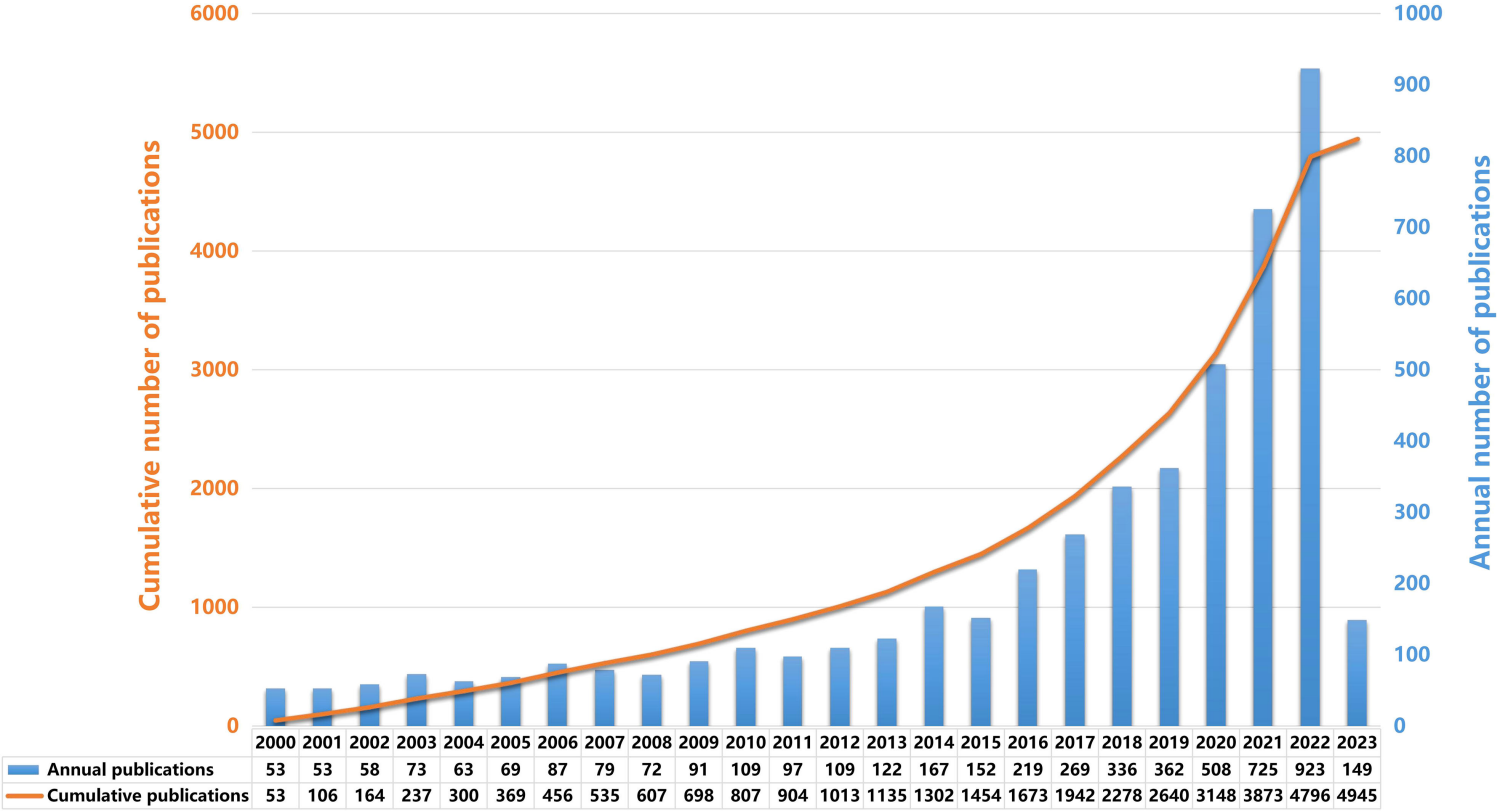
Temporal distribution plots of annual and cumulative publications. The blue bars signify the annual count of publications, while the cumulative number of publications is denoted by the orange line chart.

A total of 4910 records met the search criteria. A bar graph in **Figure 2** depicts the temporal distribution of annual publications by year. From 2000 to 2015, there was a gradual yet consistent rise in the annual volume of publications. Despite this steady growth, the number of annual publications did not surpass 200 at any point during this period. It is evident that this field garnered relatively minimal attention from researchers during that period. However, starting from 2015 to 2019, there was a more obvious upward trend in the annual publication volume, as demonstrated in the figure. In 2016, the annual publication count reached 219 in this field, marking the first instance of surpassing the 200 threshold. However, starting from 2019 to 2022, there was the most conspicuous upward trend in the annual publication volume, as demonstrated in the figure. The cumulative number of publications is depicted as a line chart. From 2000 to 2015, there was a modest increase in the publication volume, followed by a more pronounced rise from 2015 to 2022. The line chart illustrates the ascendance of this field as a research hotspot from 2019 onwards. In 2022, the annual publications soared to 923, contributing to a cumulative total of 4796. While current projections suggest that the peak of annual publications in this field could be attained in 2023, it remains plausible that this zenith has yet to be reached.

### 3.2 Geographical distribution of publications

**Figure 3:**
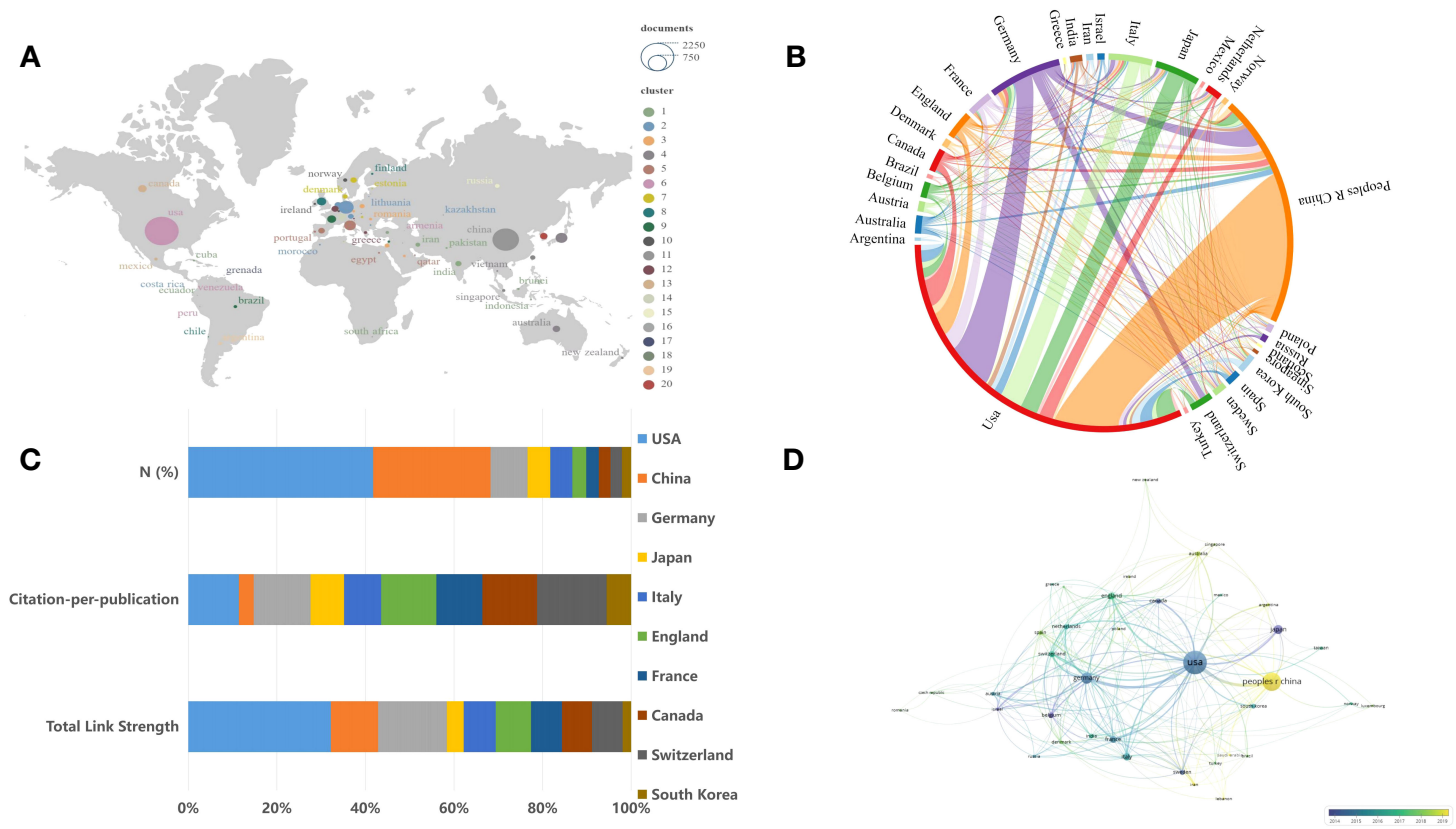
The global distribution of publications and international cooperation. **(A)** The global distribution of publications is shown, with the circle size proportional to the number of publications for each country. **(B)** Analysis of international collaboration among different countries/regions is presented. Links between countries/regions represent cooperative relationships, with thicker lines indicating stronger collaborations. **(C)** The top 10 most productive countries are listed. **(D)** An overlay visualization map of country analysis conducted using VOSviewer is shown. Each node denotes a country, with larger nodes representing more publications. Node colors indicate the range of publication dates for each country, with a gradient from purple (earliest) to yellow (most recent). The size of the node represents the number of publications in that country.

The distribution and comparison of immunotherapy for glioma research across different countries and regions can be elucidated through relevant statistics. From 2000 to 2023, a total of 79 countries or regions undertook studies in this particular field. **Figure 3A** provides a global overview of these studies, with the circle size directly proportional to the number of publications from each country. A chordal graph showed the analysis of international collaboration among various countries (**Figure 3B**). **Table 1** itemizes the top ten countries in terms of research productivity over this 13-year period. Between the years 2000 and 2023, both USA and China displayed significant activity in the given field of study. USA led in terms of research output, publishing 2180 studies that constituted 44.4% of the total research in the field. China followed with 1384 articles, comprising 28.2% of the total output. Meanwhile, Germany and Japan contributed 8.9% and 5.4% respectively, corresponding to 436 and 267 articles. We could intuitively observe that USA and China collaborated with most countries/regions in this field. Among all countries, USA collaborated most extensively with China, followed by Germany and Canada. The top five countries by total link strength were the USA (1053), Germany (508), China (348), England (260), and Italy (237). USA possesses the greatest total link strength, indicative of active collaboration with other nations in the field of immunotherapy for glioma, as corroborated by the chordal graph. However, the citation-per-publication ratio in USA is lower than that in Switzerland (**Figure 3C**). USA also dominates in citations, with China significantly lagging. Notably, although China has the second-highest number of papers, its citation-per-paper ratio is relatively low. Nevertheless, China has shown promising trends in international collaboration as evidenced by **Figure 3B**. However, it is essential to note that China’s low average cited number could be attributed to its comparatively shorter publication time. Since 2019, China has emerged as the country with the largest number of publications in this field, as illustrated in **Figure 3D**. This trend underscores China’s considerable potential and growing prominence within this area of research.

**Table 1:** The top 10 countries with the highest productivity.

Country	Publications	N (%)	Citations	Total Link Strength^a^
USA	2180	44.4%	104,474	1053
China	1384	28.2%	19,358	348
Germany	436	8.9%	23,509	508
Japan	267	5.4%	8468	124
Italy	261	5.3%	9167	237
England	163	3.3%	8509	260
France	148	3.0%	6477	228
Canada	137	2.8%	7080	222
Switzerland	137	2.8%	9038	226
South Korea	108	2.2%	2496	63

a In VOSviewer, total link strength represents the sum of links between a given node and all other nodes, indicating the node’s interactions. Link strength is a nonnegative number, with zero value denoting no links to other nodes.

### 3.3 Analysis of major institutions

**Figure 4:**
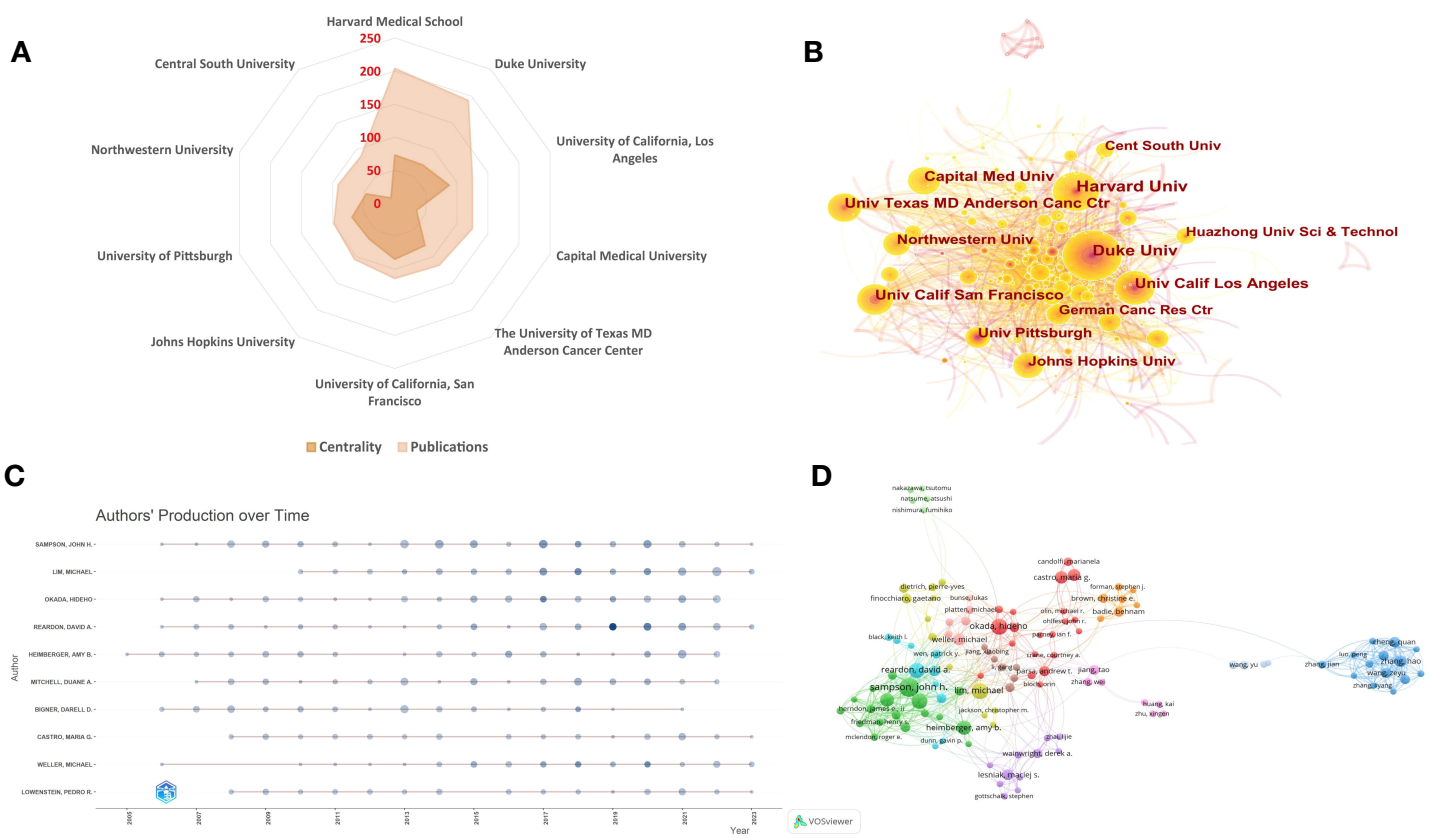
Visualization analysis of institutions and authors. **(A)** A radar map depicts the top 10 most productive institutions. **(B)** The co-occurrence map of research institutions is shown. The visual network map of institutions was generated using CiteSpace. Each node signifies an institution, with size proportional to publication output. Lines between nodes denote cooperation, with thicker lines indicating closer collaborations. The different colors indicate the year when papers were published. Node and line colors represent different time intervals, with lighter colors indicating more recent years. Nodes with high centrality (>0.1) are denoted by purple rings. **(C)** The top 10 authors’ production over time is shown. Circle size represents number of publications, with larger circles denoting more publications. Circle color shade indicates total citations for that year, with darker colors representing more citations. **(D)** A CiteSpace visualization map shows authors with at least 12 glioma immunotherapy papers. Circle size denotes number of papers. Lines between circles represent collaborations, with authors in the same color cluster exhibiting stronger co-authorship.

Citespace was used for the analysis of the main institutions. In total, 4910 bibliographies were published by 133 different institutions. The top ten most productive institutions are listed in **Table 2**. According to the analysis, the most prolific institution was Harvard Medical School (204 publications), followed by Duke University (192 publications), University of California, Los Angeles (125 publications) and Capital Medical University (125 publications). The University of Texas MD Anderson Cancer Center ranked fifth in the number of publications (**Figure 4A**). These institutions exhibited close link strengths. The University of California Los Angeles, University of California San Francisco, and University of Texas MD Anderson Cancer Center had the highest centralities, as denoted by the purple rings in **Figure 4B**, indicating their prominent influence. This maybe be associated with their early years of initiation. However, there is room for improvement in cooperation between other institutions. Among the top ten institutions, eight were from USA, two from China, corresponding to the results of the national and regional distribution. However, the centralities of top publications institutions including Capital Medical University in China, Northwestern University in USA and Central South University in China are 35, 46 and 10 respectively, which indicates their relatively weaker connections with other institutions. Thus, it is advised that institutions with the highest publication volumes, such as those mentioned above, should aim to strengthen international cooperation beyond their current academic circles. Potential approaches include attending international academic conferences in different countries and establishing communication with a wider network of researchers across diverse institutions.

**Table 2:** Top 10 most productive institutions.

Rank	Organization	Country	Publications	Centrality	Year of Initiation
1	Harvard Medical School	USA	204	73	2001
2	Duke University	USA	192	72	2000
3	University of California, Los Angeles	USA	125	88	2003
4	Capital Medical University	China	125	35	2012
5	The University of Texas MD Anderson Cancer Center	USA	116	79	2008
6	University of California, San Francisco	USA	114	85	2003
7	Johns Hopkins University	USA	105	67	2000
8	University of Pittsburgh	USA	98	69	2000
9	Northwestern University	USA	91	46	2014
10	Central South University	China	88	10	2020

### 3.4 Analysis by core authors

Using VOSviewer, we also analyzed information on authors in this research field. Of the initial 24,573 authors, we established a threshold criterion of a minimum of 12 publications per author, thereby narrowing the pool to 139 authors for in-depth analysis. Based on these criteria, the top 10 most prolific authors were extracted and visualized (**Table 3** and **Figure 4C**). The number of published articles and total citations were the key metrics used to evaluate the productivity of core authors. Sampson, John H. Ph.D., the former dean of Neurosurgery at Duke University, the Robert H. and Gloria Wilkins Distinguished Professor, is the most prolific author in the field with 88 articles and a total of 7055 citations. His research focuses on Neuro-oncology, especially the treatment and immunotherapy of glioblastoma. He has made significant contributions to the development of intracranial tumor surgery, neuro-oncology, and immunotherapy, and under his leadership, the Department of Neurosurgery at Duke University is considered one of the leading neurosurgical centers in the world. Professor at Johns Hopkins University School, Lim, Michael, focuses on Neurosurgical oncology, skull base surgery, stereotactic radiosurgery, is second on the list with 65 papers and a total of 5219 citations in this field, followed by Okada, Hideho from University of California, San Francisco (64 publications). The extent of cooperative relationships among authors from different countries was limited (**Figure 4D**). It is evident from the figure that core authors from USA participated in most international collaborations and served as the center of various subgroups, such as Sampson, John H., Okada, Hideho, and Lim, Michael. On the other hand, researchers from some countries within their own countries, indicating that some researchers in this field should strengthen their international cooperation efforts.

**Table 3:** The top 10 most prolific core authors.

Author	Documents	Citations	Total Link Strength	Country	Institution	Research Focus
Sampson, John H.	88	7055	269	USA	Duke University	Neuro-oncology, especially the treatment and immunotherapy of glioblastoma
Lim, Michael	65	5219	71	USA	Johns Hopkins University	Neurosurgical oncology, skull base surgery, stereotactic radiosurgery
Okada, Hideho	64	4290	73	USA	University of California, San Francisco	Immunotherapies for the treatment of malignant gliomas and other central nervous system tumors
Mitchell, Duane A.	57	4239	187	USA	University of Florida College of Medicine	Development of new treatment strategies to improve survival rates in patients with neuro-oncology, especially pediatric and adult glioblastomas
Reardon, David A.	57	5276	160	USA	Harvard Medical School, Dana-Farber Cancer Institute	Neuro-oncology, especially the treatment of glioblastoma
Heimberger, Amy B.	53	4339	89	USA	MD Anderson Cancer Center, Texas	Neuro-oncology, especially the treatment and immunotherapy of glioblastoma
Bigner, Darell D.	47	4556	190	USA	Duke University	Neuro-oncology, especially the pathology and treatment of glioblastoma
Castro, Maria G.	41	1568	65	USA	University of Michigan	Gene therapy for the treatment of neuro-oncology
Lowenstein, Pedro R.	39	1523	63	USA	University of Michigan	Gene therapy and etiological research of neuro-oncology
Weller, Michael	39	4006	54	Switzerland	University Hospital Zurich	Neuro-oncology, especially the treatment of glioblastoma

### 3.5 Distribution of journals

**Figure 5:**
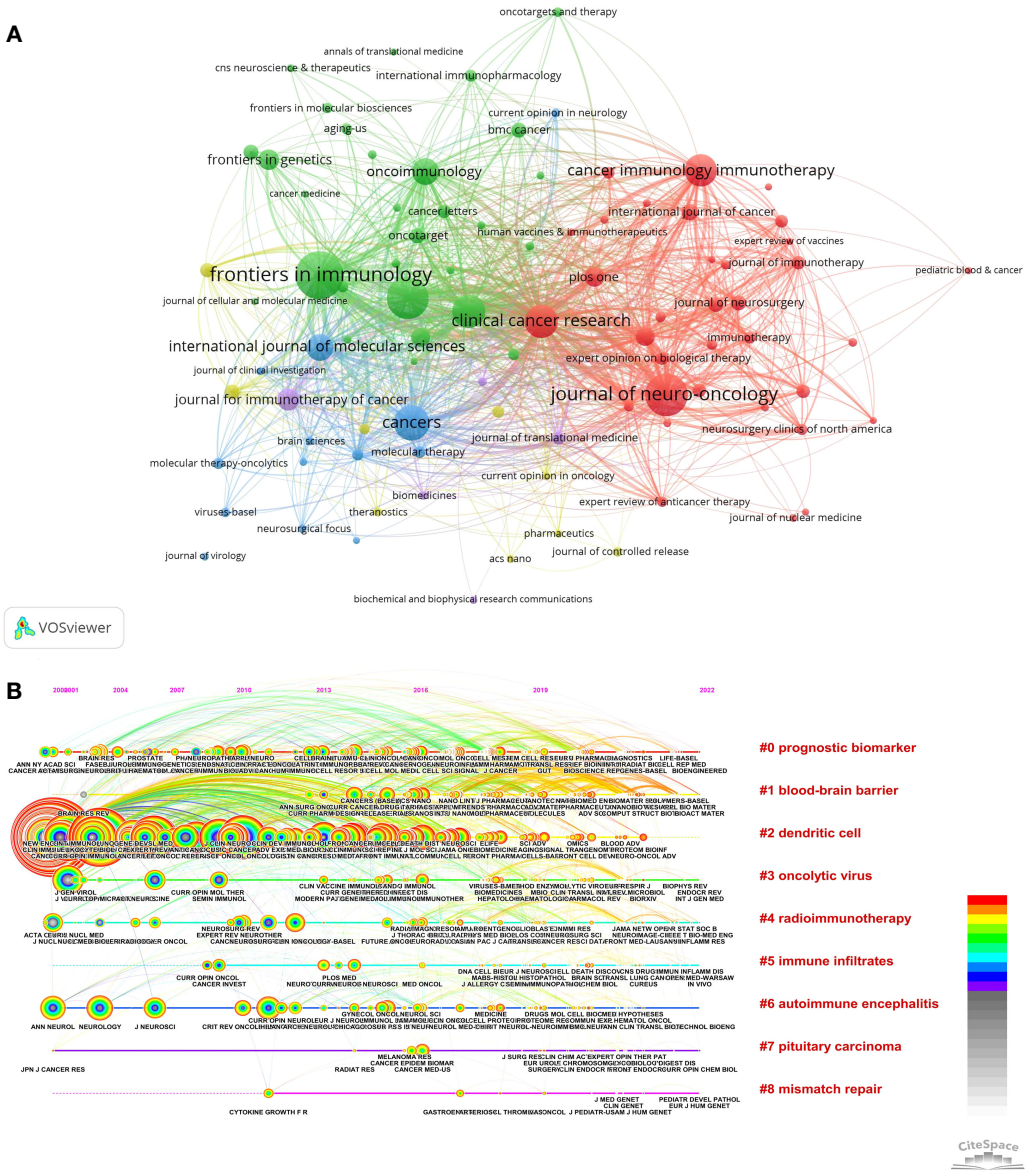
Analysis of journals. **(A)** Network visualization map, generated by VOSviewer, depicts journal co-occurrence analysis. Each node symbolizes a journal, with its size proportional to the number of publications within that journal. Each node’s color corresponds to a cluster, with different hues representing distinct clusters. The density of links between two nodes indicates the co-authorship relationship among journals. **(B)** The timeline view for co-cited journals related to immunotherapy for glioma by Citespace. The size of each node corresponds to the number of co-citations for the respective journals, while the connecting curves between nodes signify co-citation relationships. The color on the right, red, is closer to the present, and purple is older.

By analyzing the publication sources in this field, we identified the core journals. Our analysis identified the top ten journals characterized by the highest publication count, accompanied by pertinent details regarding each (**Table 4**). The most prolific journal was *Frontiers in Immunology* (impact factor 8.787) with 215 publications, followed by *Journal of Neuro-oncology* (177 publications, IF 4.506) and *Frontiers in Oncology* (173 publications, IF 5.738). The impact factors of these top journals ranged from 4.506 for *Journal of Neuro-oncology* to 13.801 for *Clinical Cancer Research*. *Clinical Cancer Research* had the most citations (9701), followed by *Neuro-oncology* (8624) and *Journal of Neuro-oncology* (4895). *Clinical Cancer Research* also had the highest average citation per publication (80.17) and total link strength (3376), indicating its substantial contributions to glioma immunotherapy publications. Collectively the top 10 journals accounted for 1291 publications, representing 26.3% of the total. **Figure 5** represents two distinct network visualization maps. **Figure 5A**, generated by VOSviewer, presents the network visualization of journals. Only journals with a minimum of 12 documents were included in the visualization. The map contains 90 nodes, 2,566 links, and 5 clusters. VOSviewer cluster analysis delineated five clusters, represented by the five colors in the figure (red, green, blue, yellow, purple).

**Table 4:** Top 10 journals in terms of number of publications published.

Journal	Publications	Citations (WOS)	Average citation/publication	Impact factor (2021)	Total Link Strength
*Frontiers in Immunology*	215	3505	16.30	8.787	2443
*Journal of Neuro-oncology*	177	4895	27.66	4.506	1932
*Frontiers in Oncology*	173	2370	13.70	5.738	1643
*Cancers*	134	1747	13.04	6.575	1798
*Clinical Cancer Research*	121	9701	80.17	13.801	3376
*Neuro-oncology*	119	8624	72.47	13.029	3015
*Cancer Immunology Immunotherapy*	114	4278	37.53	6.630	1363
*International Journal of Molecular Sciences*	87	1291	14.84	6.208	914
*Oncoimmunology*	86	2917	33.92	7.723	1176
*Journal for Immunotherapy of Cancer*	65	1333	20.51	12.469	598

WOS, Web of Science.

Cluster 1 was represented by *Frontiers in Immunology*, which as the official journal of the International Union of Immunological Societies (IUIS) and the top cited journal in immunology, is at the forefront of basic, translational and clinical immunology research.

Cluster 2 was represented by *Journal of Neuro-oncology*. As the name of this journal, it is a multi-disciplinary journal encompassing basic, applied, and clinical investigations in all research areas as they relate to cancer and the central nervous system. It provides a single forum for communication among neurologists, neurosurgeons, specialists, and laboratory-based oncologists conducting relevant research.

Cluster 3 was represented by *Frontiers in Oncology*, a broad-scope multidisciplinary journal covering all areas of cancer research including carcinogenesis, tumor progression, and bridging basic science with clinical applications to advance public knowledge and improve cancer diagnosis, therapeutics, and management.

Cluster 4 was represented by *Cancers*. It is an international, peer-reviewed open access journal in the field of oncology. It seeks to motivate scientists to share their experimental and theoretical outcomes with comprehensive detail. A distinctive attribute of this journal is its receptiveness to studies that convey meaningful, albeit negative, results.

Cluster 5 was represented by *Clinical Cancer Research*. This journal is dedicated to the publication of articles centered on pioneering clinical and translational research, thereby connecting laboratory work with clinical practice. Covered topics encompass targeted therapies, advancements in pharmacogenetics and pharmacogenomics, immunotherapy, etc.

**Figure 5B**, generated by Citespace, shows the timeline view of co-cited journals in glioma immunotherapy. This temporal visualization depicts evolving trends and hotspots in the field. A co-citation relationship occurs when two journals are cited together in a publication. High co-citation counts indicate journals producing high-impact work. Co-citation analysis was performed on 187 journals from 4910 publications. Larger nodes denote frequently cited top journals like *Cancer Research*, *Clinical Cancer Research*, and *Neuro-oncology*. The clustering timeline revealed the top nine clusters as: “Prognostic biomarker”, “Blood-brain barrier”, “Dendritic cell”, “Oncolytic virus”, “Radioimmunotherapy”, “Immune infiltrates”, “Autoimmune encephalitis”, “Pituitary carcinoma” and “Mismatch repair”. In recent years, “Blood-brain barrier”, “Prognostic biomarker” and “Dendritic cell” demonstrated high influence. Prognostic biomarkers play a crucial role in the management of glioma by informing therapeutic strategies. However, the effectiveness of drug-based treatments for glioma is often constrained by the restrictive properties of the blood-brain barrier. The significance of dendritic cells in glioma research has become increasingly evident post-2000, as substantiated by findings from eminent publications such as *Cancer Research*, *Clinical Cancer Research*, *Neuro-oncology*, and *New England Journal of Medicine*. The citation burst is a sudden and significant increase in the number of citations a particular reference receives over a specific time period, indicating that the topic or method presented in the reference has gained heightened attention or relevance. The color of each link corresponds to the date when the two papers were first co-cited. Node and link colors denote distinct years, with warmer hues indicating more recent publication dates. Nodes possessing a high centrality (greater than 0.1) are encircled in purple, while those experiencing citation bursts are marked with red dots. Each horizontal line is arranged chronologically from left to right. On the right side, clustering labels are presented based on the topic algorithm. Additionally, the number of included publications diminishes from top to bottom.

### 3.6 Analysis of keyword

**Figure 6:**
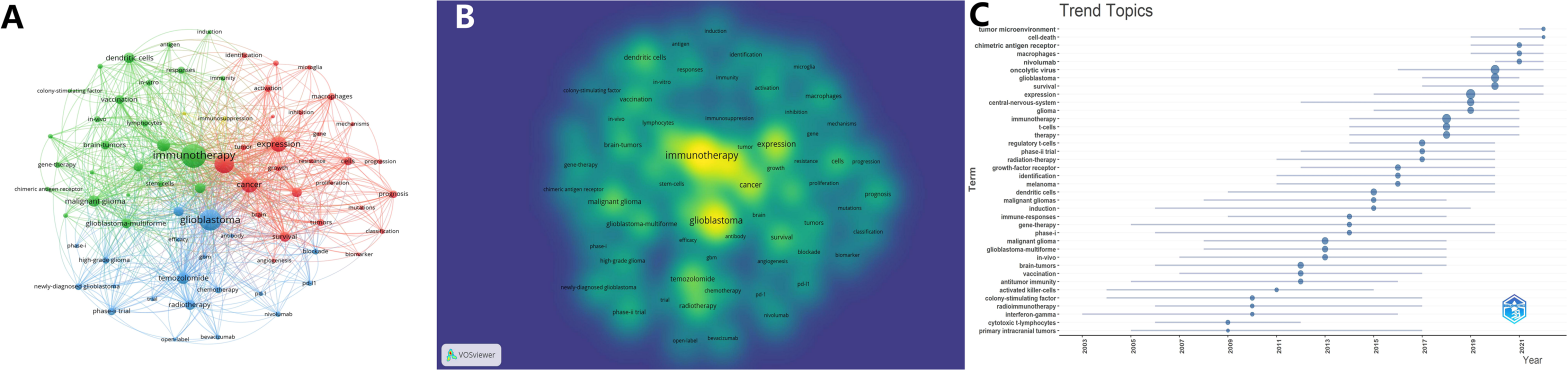
Bibliometric Aanalysis of keywords in glioma immunotherapy research papers. **(A)** Thematic network maps illustrating keyword trends in glioma immunotherapy research from January 1, 2000, to April 1, 2023. **(B)** Keyword co-occurrence density; the most prevalent keywords are highlighted in yellow. **(C)** Temporal trends in topics: the circle size corresponds to the number of publications, with larger circles indicating a higher number of documents.

Keywords represent the essence of an article. By analyzing these keywords, one can discern the central theme of the article and identify prevailing trends within specific research domains. We employed overlay visualizations of both network and density map to analyze the co-occurrence of these keywords. From an initial compilation of 12,890 keywords, we established a minimum occurrence threshold of 90, a parameter designed to focus on the most relevant and frequently occurring terms, ultimately refining our focus to the 76 most frequently occurring keywords for detailed analysis and divided them into three distinct clusters (**Figure 6A**), which was made the density of co-occurrence according to the frequency of keywords (**Figure 6B**). In the network graphs, the keywords were primarily classified into three categories. The first cluster, highlighted in green, represented research related to glioma fundamental or mechanistic research. This group included a range of cell types and molecules that play a role in the immune system. The second cluster, highlighted in blue, encompassed terms commonly associated with clinical trials, such as phase I, phase II trial, open-label, and treatments for glioma, such as chemotherapy, nivolumab, bevacizumab and radiotherapy. The third cluster, denoted by red, predominantly centers on investigating the roles and functions of immune cells associated with gliomas, with keywords such as macrophages, activation, inhibition, mutations, and proliferation. Based on the density of co-occurrence, the keywords most frequently used are ‘immunotherapy’ (2180 occurrences), ‘glioblastoma’ (1756 occurrences), and ‘expression’ (1014 occurrences). By observing the trend topics over time (**Figure 6C**), we noticed that they were in order of knowledge of glioma immunotherapy over time from shallow to deep, reflecting the transformation of research focus and development process in this field. These emerging topics suggest a change in focus towards a deeper understanding of the complex interplay between tumors and the immune system. Notably, some keywords in the top -left corner of the Figure 6C, such as tumor microenvironment, cell-death, chimetric antigen receptor, macrophages and nivolumab, suggesting a discernible shift in the emphasis and orientation of future research endeavors.

#### 3.6.1 Implications of emerging keywords for glioma immunotherapy

Tumor microenvironment (22) - The tumor microenvironment refers to the surrounding cellular environment in which a tumor exists. This includes immune cells, blood vessels, signaling molecules, the extracellular matrix, and more. Understanding and potentially manipulating the tumor microenvironment is key for improving immunotherapies against gliomas and other cancers. Active communication between glioma cells and adjacent healthy cells, as well as the surrounding immune environment, facilitates oncogenic processes and contributes to the formation of glioma stem cells, which in turn promote resistance to therapy.

Cell death (23) - There are different types of regulated cell death that can be induced in tumor cells, including apoptosis, necroptosis, and pyroptosis. Finding ways to selectively induce tumor cell death while sparing normal healthy brain cells will be important for developing effective immunotherapies. Targeting cell death pathways in tumor cells can also release antigens and stimulate an anti-tumor immune response. In glioblastoma patients, Enriched ferroptosis were associated with advanced malignancy, poor prognostic outcomes, and increased immunosuppression. Although enhanced ferroptosis stimulated the activation and infiltration of immune cells, it concurrently diminished their antitumor cytotoxic efficacy. Moreover, tumor-associated macrophages (TAM) were implicated in the immunosuppressive effects mediated by ferroptosis.

Chimeric antigen receptor (CAR) T cells (24) - CAR T cell therapy is a promising new approach for glioma immunotherapy. It involves engineering a patient’s own T cells to express a receptor that recognizes a tumor antigen. This allows the CAR T cells to selectively bind to and kill the tumor cells. Optimizing the antigen targets, T cell engineering, and getting the cells to traffic to the tumor site will be important areas for improving CAR T therapy against gliomas. The clinical trial highlighted the potential efficacy of this therapeutic approach for patients with H3K27M-mutated diffuse intrinsic pontine glioma (DIPG) or spinal cord diffuse midline glioma (DMG) (25).

Macrophages (26) - Tumor associated macrophages are a major immune cell population in the glioma microenvironment. Finding ways to repolarize them from a tumor-promoting to a tumor-killing phenotype could greatly enhance immunotherapies. Strategies include depleting certain macrophage subsets and activating them through immune stimulants. The predominant non-neoplastic cells within the tumor milieu are TAMs, originating either peripherally or from brain-intrinsic microglia. These TAMs form a supportive stroma that facilitates neoplastic cell proliferation and invasion. Recruited to the glioma environment, TAMs exhibit immunomodulatory functions and secrete a diverse array of growth factors and cytokines in response to those produced by cancer cells. Through these reciprocal interactions, a unique tumor microenvironment is established, presenting novel avenues for therapeutic targeting.

Nivolumab (27) - Nivolumab is an immune checkpoint inhibitor antibody used in cancer immunotherapy. It blocks PD-1 on T cells to prevent inhibition of the anti-tumor immune response. While nivolumab hasn’t shown much promise yet against gliomas, combining it with vaccines, CAR T cells, or other immunotherapies may improve its efficacy. Determining the right combination and timing of treatments will be key. A prior study demonstrated that Nivolumab effectively inhibited the expansion of regulatory T cells (Tregs) induced by PD-L1 (28).

In summary, further understanding the glioma tumor microenvironment, exploiting cell death pathways, improving CAR T cell engineering, targeting immunosuppressive macrophages, and combining checkpoint inhibitors like nivolumab with other immunotherapies represent key areas for advancing near-future glioma immunotherapies.

### 3.7 Analysis of cited and co-cited references

Citation and co-citation analyses of the literature facilitate an understanding of the field’s seminal research and provide direction for future scholarly endeavors. This study encompassed 145,516 cited references, with 49 publications receiving over 171 citations each. We utilized bibliometrix functions to extract the number of citations for each publication, and the top ten citations are presented in **Table 5**. The most frequently cited articles, published in *Acta Neuropathologica* in 2016. Notably, two of the top ten cited articles were reviews, all of which were published in high-impact journals and authored by leading researchers in the field.

**Table 5:** The top 10 most cited publications.

Paper	DOI	Type	Citation	Burst	Centrality
Louis DN, 2016, ACTA NEUROPATHOL (22)	10.1007/s00401-016-1545-1	Review	332	90.5	8
ORourke DM, 2017, SCI TRANSL MED (26)	10.1126/scitranslmed.aaa0984	Article	327	59.2	36
Cloughesy TF, 2019, NAT MED (27)	10.1038/s41591-018-0337-7	Article	312	75.8	43
Brown CE, 2016, NEW ENGL J MED (28)	10.1056/NEJMoa1610497	Article	280	78.3	26
Lim M, 2018, NAT REV CLIN ONCOL (29)	10.1038/s41571-018-0003-5	Review	272	61.7	15
Weller M, 2017, LANCET ONCOL (30)	10.1016/S1470-2045(17)30517-X	Article	249	44.8	41
Reardon DA, 2020, JAMA ONCOL (9)	10.1001/jamaoncol.2020.1024	Article	237	95.3	27
Ahmed N, 2017, JAMA ONCOL (31)	10.1001/jamaoncol.2017.0184	Article	212	38.1	25
Nduom EK, 2016, NEURO-ONCOLOGY (32)	10.1093/neuonc/nov172	Article	198	44.4	32
Schalper KA, 2019, NAT MED (33)	10.1038/s41591-018-0339-5	Article	186	47.0	40

**Figure 7:**
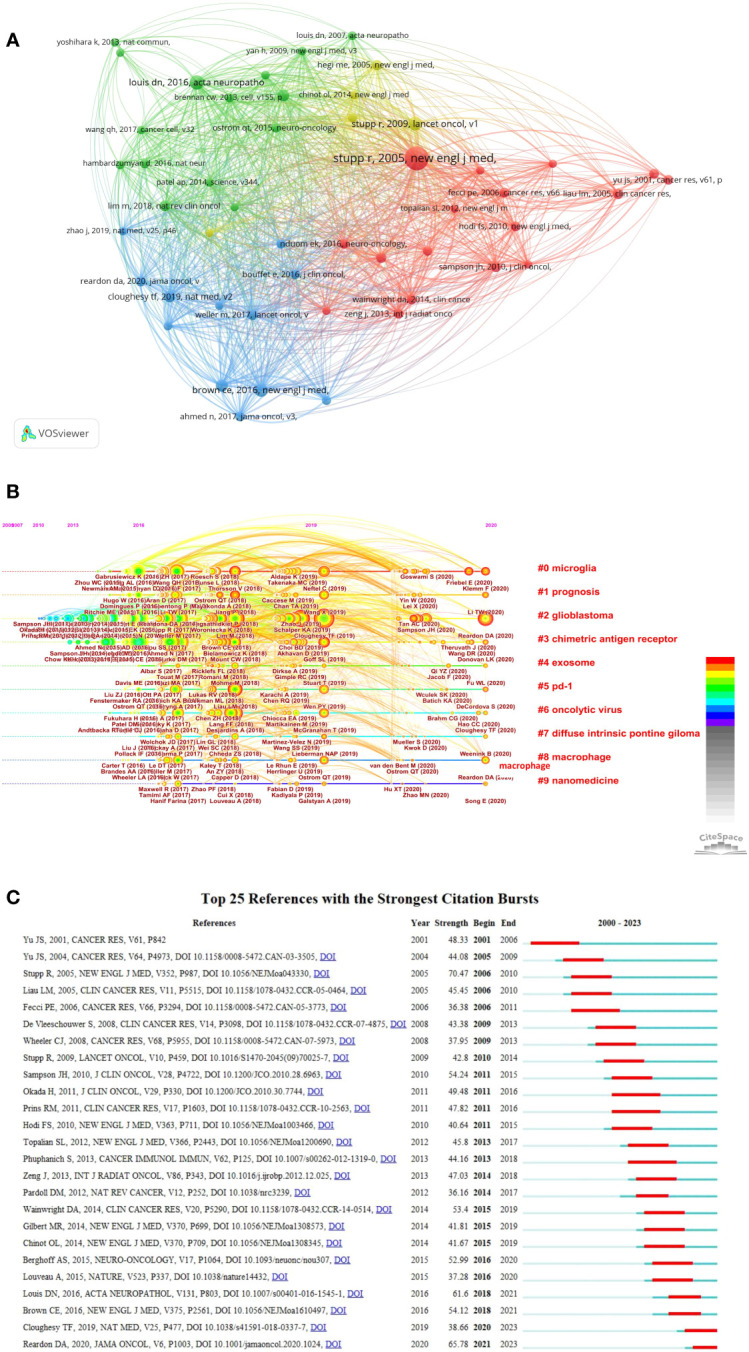
Visualization analysis of cited and co-cited references. **(A)** Cluster analysis of co-cited references; **(B)** Timeline visualization of co-cited references cluster analysis result from January 1, 2000 to April 1, 2023; **(C)** The top 25 references exhibit the most pronounced co-citation bursts. The strength of a citation burst is a metric that quantifies the intensity of the burst during the period in which it occurs. Higher strength values indicate a more significant increase in citations over a short period, suggesting greater impact or influence in the field during that time.

Utilizing the co-citation network, we conducted a reference burst analysis. **Figure 7** displays the top 25 co-cited references exhibiting the most pronounced citation bursts. As depicted in **Figure 7A**, references (29–31) emerged as the three most co-cited. The timeline view of the co-citation references offers a visual representation, elucidating the temporal evolution of research hotspots within this domain. As inferred from **Figure 7B**, among the 10 clusters, glioblastoma emerged as the earliest research focal point in this domain and currently remains the predominant research hotspot. Microglioma, chimetric antigen receptor, exosome, PD-1, oncolytic virus, macrophage and nanomedicine have been subjects of significant interest to researchers since 2013. **Figure 7C** presents a summary of the top 25 references with the most significant citation bursts. The onset of the reference citation burst in this study can be traced back to 2001, attributed to the paper authored by J. S. Yu (32). The most recent citation burst was identified in 2021 and continues to the present.

## 4 Discussion

In this era of exponential information growth, maintaining awareness of research hotspots, keeping abreast of the latest findings, and sustaining a leading position in one’s field is challenging. Bibliography retrieval and knowledge management are therefore routine tasks for scientific researchers. Unlike systematic reviews or meta-analyses, bibliometric analysis uniquely enables summarization of the development of specific research areas and identification of emerging research hotspots. This is the first study to utilize bibliometric methods to summarize the application and development of glioma immunotherapy over the past 23 years, delineate the developmental trends, and predict future research hotspots in this field.

In this bibliometric analysis, we used Microsoft Excel, VOSviewer, CiteSpace and bibliometrix package in R for bibliometric analysis and network visualization, and comprehensively understood the relevant literature, research hotspots and trends of immunotherapy for glioma. The search was completed on April 1, 2023, with a final bibliometric analysis and visualization of 4910 publications from January 1, 2000 to April 1, 2023.

### 4.1 Publications over time

The annual publication output fluctuated over the years without a discernible growth trend until 2019, which mirrored the publication count of 2017. However, between 2020 and 2022, there was a notable upward trend, with an annual output surpassing 250 papers. **Figure 2** presents a line chart depicting the cumulative publication count. Between 1999 and 2019, the cumulative number of publications grew at a modest pace. However, from 2020 to 2022, this growth accelerated, reaching nearly 3,000 publications by 2022, suggesting that the peak might either occur in 2022 or is yet on the horizon.

From 2000 to 2015, there was a consistent increase in annual publications, yet the count remained below 200 throughout this period, indicating limited research focus in this field. A marked increase in annual publications was evident from 2015 to 2019. In 2016, the count exceeded 200 for the first time, reaching 219. The years 2019 to 2022 witnessed the most pronounced growth, as illustrated in the figure. The cumulative publication count, represented in the line chart, grew modestly from 2000 to 2015 but saw a steeper ascent from 2015 to 2022. By 2022, the annual count had surged to 923, accumulating to a total of 4,796 publications. Current data suggests a potential peak in annual publications by 2023, though the apex might still be forthcoming. These trends strongly indicate that immunotherapy for glioma is emerging as a focal point in neuroscience research, poised for rapid expansion. An objective analysis of publications and citations underscores the evolving scientific landscape of glioma immunotherapy.

### 4.2 Geographical distribution and major institutions

In terms of geographical distribution and major institutions, 4910 publications from 78 countries were published in terms of geographical distribution. **Table 1** presents the 10 most productive countries, with USA leading the way with 2180 publications, followed by China with 1384 publications and Germany with 436 articles. USA is also dominant in citations, however, China’s total citations were suboptimal, with the average citation per paper being the lowest among the top 10 countries/regions by productivity (**Figure 3C**), probably due to its relatively short publication time. As depicted in **Table 2**, eight of the top ten most productive organizations were from USA, further underscoring this country’s dominance in this research field (**Figure 3A**). As for the citation-per-publications, Switzerland ranked the first with 66. However, USA’s citation-per-publications is 47.9, and China’s citation-per-publications is 14, suggesting that the above countries should improve the quality of each publication so as to increase its influence. After 2019, China has gradually become the country with the largest number of publications in this field (**Figure 3D**), which also shows China’s great potential in this research field. All these indicate that Chinese scholars should further strengthen the quality of research in the future, and enhance international cooperation and exchange, so as to produce more high-quality articles.

As for cooperation between countries, USA was at the epicenter of research activity and maintained close collaborations primarily with China, Germany, Canada and England. However, most cooperation and communication were confined to North America, Europe and a handful of Asian nations. Moving forward, enhancing international and cross-boundary cooperation will be imperative, particularly with developing countries. The role of economic support and research funding in enabling scientific output cannot be ignored. Many countries may benefit from increased investment and encouragement for research, which could facilitate their emergence as significant contributors in this field.

### 4.3 Analysis of core authors

In the analysis of core authors, nine of the top ten were from USA, underscoring the country’s preeminence in this research area. This finding aligns with the results from the institutional analysis. Among these authors, John H. Sampson led in terms of publication count, closely followed by Michael Lim and Hideho Okada, with 65 and 64 papers, respectively. Interestingly, David A. Reardon, despite having fewer publications, ranked second in total citations, only behind Sampson, attesting to his significant influence in the domain. **Figure 4D** illustrates the central roles of John H. Sampson, Hideho Okada, Michael Lim, and David A. Reardon, as they appear to be key connectors across multiple research clusters, which may account for their elevated citation metrics. Conversely, **Figure 6D** highlights existing silos in collaboration, with many research teams predominantly co-authoring within their own countries. This observation underscores the need to bolster international collaborations, fostering knowledge transfer, resource pooling, and innovative breakthroughs, which in turn can expedite research advancements and therapeutic innovations.

### 4.4 Distribution of journals

Identifying influential journals and conducting journal co-citation analysis can provide researchers with valuable reference information to guide selection of appropriate target journals when searching literature or submitting manuscripts. Total citations and impact factor (IF) (33) are two key metrics for evaluating the academic status of journals. In terms of core journals in this field, **Table 4** shows that Clinical Cancer Research has the largest total number and IF, which may be the most influential journal in the field of immunotherapy for glioma. Focusing on these relatively influential core journals will help to understand the frontier of immunotherapy for glioma. The impact factors ranged from 5.738 to 13.801, indicating that glioma immunotherapy articles are publishable in high-impact journals. Frontiers in Immunology had the most published articles, suggesting it is the primary target journal for publications in this field.

Journal co-citation analysis offers insights into the connections between different research findings (34). *Clinical Cancer Research, Neuro-oncology*, and *Frontiers in Immunology* had the top three total link strengths, indicating that glioma immunotherapy papers in these journals are highly cited. Researchers are advised to closely follow findings published in these sources to stay abreast of the latest advances.

### 4.5 Analysis of keywords

Co-occurrence analysis of keywords is a bibliometric technique commonly used to identify popular research topics and track the evolution of focus areas, thereby elucidating research hotspots. As depicted in **Figure 6B**, “immunotherapy” and “glioblastoma” were the most frequent keywords, aligning with the topic of this study.

In the network graphs, the keywords were primarily classified into three categories. The first cluster, highlighted in green, represented research related to glioma fundamental or mechanistic research. This group included a range of cell types and molecules that play a role in the immune system. The second cluster, highlighted in blue, encompassed terms commonly associated with clinical trials, such as phase I, phase II trial, open-label, and treatments for glioma, such as chemotherapy, nivolumab, bevacizumab and radiotherapy. The third cluster, denoted by red, predominantly centers on investigating the roles and functions of immune cells associated with gliomas, with keywords such as macrophages, activation, inhibition, mutations, and proliferation.

According to the trend topics, such as tumor microenvironment, cell-death, chimetric antigen receptor, macrophages and nivolumab, denoting a shift in research emphasis and future trajectory to some degree. The tumor microenvironment underlies acquired resistance to colony stimulating factor-1 receptor inhibition in gliomas (35). Cell death is a normal and regulated process that occurs in organisms (36, 37). There are different types of cell death, including apoptosis (programmed cell death), necrosis, autophagy, and others. Immune cells like cytotoxic T cells and NK cells can be activated to induce apoptosis in tumor cells by releasing perforins, granzymes, and other cytotoxic molecules. Checkpoint inhibitor drugs block signals that prevent T cell activation and help T cells stay active against tumor cells. Other immunotherapies enhance T cell or NK cell activity. Some glioma cells develop defects in apoptosis pathways, allowing them to resist cell death. Understanding the regulators and evaders of cell death in glioma cells will be important for designing effective combination immunotherapies. Chimeric antigen receptor (CAR) is a specific receptor expressed on the membrane of T cells after artificial gene editing, which can recognize tumor-specific antigens and activate T cells after introduction into the body (38). Its principle of treating glioma has attracted much attention.

### 4.6 Citation and co-citation analysis of references

Citation and co-citation analysis of references are key techniques in bibliometric studies to identify influential literature and assess research evolution, thereby predicting frontiers of knowledge advancement. Highly cited articles generally represent high-quality, innovative research with substantial impact in a given field. **Table 5** lists the top 10 most highly cited publications, which have all exerted significant influence in this field. Specifically, the most frequently cited articles, published in *Acta Neuropathologica* in 2016, the study represented the first WHO classification of CNS tumors to incorporate molecular parameters alongside histology in defining many tumor entities, thus establishing a framework for molecular era diagnostics (29). Another article with 327 citations was published in *Science Translational Medici*ne in 2017 by ORourke DM et al. His study reported initial experience with CAR-T cells for recurrent glioblastoma, suggesting that while intravenous infusion mediated on-target brain activity, efficacy of Epidermal Growth Factor Receptor variant III (EGFRvIII)-directed approaches may be improved by overcoming adaptive tumor microenvironment changes and addressing antigen heterogeneity (39).

Burst detection is an algorithm that captures sudden surges in the popularity of references or keywords within a defined timeframe, serving as an effective approach for identifying hot topics and research fronts. The article with the highest burst strength (70.47) was published in 2005 by Roger Stupp et al. (31), demonstrating that adding temozolomide to radiotherapy for newly diagnosed glioblastoma conferred clinically meaningful and statistically significant survival benefits with minimal toxicity. Many top articles continue to be highly cited, indicating that glioma immunotherapy will likely remain a research hotspot in coming years.

This bibliometric analysis holds distinct implications for both physicians and patients:

For physicians:

Knowledge advancement: Our study highlights the seminal papers and leading authors in glioma immunotherapy, equipping physicians with the latest pivotal research and evidence-based methodologies.

Collaboration insights: By identifying the primary contributing nations and institutions, we provide physicians with a window into potential hubs of excellence and collaborative ventures.

Treatment overview: Our keyword and thematic evaluations present a concise overview of the shifting treatment landscape, elucidating emerging therapeutic approaches and anticipated challenges.

For patients:

Informed choices: By spotlighting influential research, we empower patients with insights into the most recent and significant advancements in glioma immunotherapy, aiding them in treatment-related decisions and dialogues with healthcare professionals.

Setting expectations: Recognizing the research trajectories and key contributors allows patients to foster realistic anticipations regarding the prospective outcomes and trajectories of glioma immunotherapy.

Decisional empowerment: Familiarity with the forefront institutions in glioma immunotherapy research can guide patients in their choices concerning healthcare consultation or clinical trial participation.

### 4.7 Strengths and limitations

This study is the first to use bibliometric analysis to analyze the research trend of immunotherapy for glioma. Bibliometric analysis is more comprehensive than literature review, and the visualization effect is better. Secondly, this study uses multiple softwares: Microsoft Excel, Microsoft charticulator, VOSviewer 1.6.18, Citespace 5.7R3, bibliometrix package in R and online tool (https://www.scimagojr.com/). To better carry out literature data processing, bibliometric analysis and visualization.

Nonetheless, the study has some limitations. First of all, Some relevant articles may have only been categorized under “complementary medicine”, “alternative medicine”, or “translational medicine” in the WOS database, possibly resulting in their exclusion. Thus, our study may not fully encompass all literature pertinent to this research area. Second, only data obtained from the WoSCC database were included in this study and we excluded non-English papers. Other databases need to be analyzed in future studies. Moreover, the bibliometric techniques used here are limited to metadata rather than full text analysis. Therefore, we may have missed critical insights only present in the full articles, such as author perspectives and outlook on the field. Finally, the database is constantly updated and only relevant records from January 1, 2000 to April 1, 2023 are considered in this study. Because the journal name is too long, some journal name overlap in **Figure 5**. Consequently, there may be discrepancies between this bibliometric analysis and the actual state of the literature on immune checkpoint inhibitors for glioma.

## 5 Conclusion

In our pioneering bibliometric analysis covering glioma immunotherapy publications from 2000 to 2023, we observed a significant surge in research activity, predominantly led by USA. The current hotspots in the field include CAR-T, PD-1, and nivolumab. Future studies should prioritize the identification of distinct neoantigens and prognostic tumor markers, innovate approaches to augment immune responses, and tackle challenges like glioma’s immunosuppressive tendencies and immune evasion. Simultaneously, it is imperative to ensure patient safety by addressing non-specific immunity risks associated with immunotherapy. Enhanced international collaboration is essential to progress in these research domains.

## Data availability statement

The raw data supporting the conclusions of this article will be made available by the authors, without undue reservation.

## Ethics statement

Given that the current study publicly accessible, de-identified data from investigations that had already attained ethical committee endorsement, thus negating the need for subsequent ethical clearance.

## Author contributions

H-YZ: Conceptualization, Data curation, Writing – original draft. H-YY: Investigation, Methodology, Writing – original draft. G-XZ: Software, Supervision, Writing – review & editing. X-ZJ: Data curation, Formal Analysis, Writing – original draft. GG: Supervision, Validation, Visualization, Writing – review & editing. B-JW: Funding acquisition, Project administration, Resources, Supervision, Validation, Visualization, Writing – review & editing.
